# Overexpression of Receptor Tyrosine Kinase EphB4 Triggers Tumor Growth and Hypoxia in A375 Melanoma Xenografts: Insights from Multitracer Small Animal Imaging Experiments ^‡^

**DOI:** 10.3390/molecules23020444

**Published:** 2018-02-17

**Authors:** Christin Neuber, Birgit Belter, Sebastian Meister, Frank Hofheinz, Ralf Bergmann, Hans-Jürgen Pietzsch, Jens Pietzsch

**Affiliations:** 1 Department Radiopharmaceutical and Chemical Biology, Helmholtz-Zentrum Dresden-Rossendorf, Institute of Radiopharmaceutical Cancer Research, Bautzner Landstrasse 400, 01314 Dresden, Germany; c.neuber@hzdr.de (C.N.); b.belter@hzdr.de (B.B.); s.meister@hzdr.de (S.M.); r.bergmann@hzdr.de (R.B.); 2 Department Positron Emission Tomography, Helmholtz-Zentrum Dresden-Rossendorf, Institute of Radiopharmaceutical Cancer Research, Bautzner Landstrasse 400, 01314 Dresden, Germany; f.hofheinz@hzdr.de; 3 Department Radionuclide Theragnostics, Helmholtz-Zentrum Dresden-Rossendorf, Institute of Radiopharmaceutical Cancer Research, Bautzner Landstrasse 400, 01314 Dresden, Germany; h.j.pietzsch@hzdr.de; 4 Faculty of Chemistry and Food Chemistry, School of Science, Technische Universität Dresden, 01062 Dresden, Germany

**Keywords:** Eph receptor tyrosine kinase family, Ephrin ligands, tumor microenvironment, malignant melanoma, small animal positron emission tomography, tumor angiogenesis, tumor hypoxia

## Abstract

Experimental evidence has associated receptor tyrosine kinase EphB4 with tumor angiogenesis also in malignant melanoma. Considering the limited in vivo data available, we have conducted a systematic multitracer and multimodal imaging investigation in EphB4-overexpressing and mock-transfected A375 melanoma xenografts. Tumor growth, perfusion, and hypoxia were investigated by positron emission tomography. Vascularization was investigated by fluorescence imaging in vivo and ex vivo. The approach was completed by magnetic resonance imaging, radioluminography ex vivo, and immunohistochemical staining for blood and lymph vessel markers. Results revealed EphB4 to be a positive regulator of A375 melanoma growth, but a negative regulator of tumor vascularization. Resulting in increased hypoxia, this physiological characteristic is considered as highly unfavorable for melanoma prognosis and therapy outcome. Lymphangiogenesis, by contrast, was not influenced by EphB4 overexpression. In order to distinguish between EphB4 forward and EphrinB2, the natural EphB4 ligand, reverse signaling a specific EphB4 kinase inhibitor was applied. Blocking experiments show EphrinB2 reverse signaling rather than EphB4 forward signaling to be responsible for the observed effects. In conclusion, functional expression of EphB4 is considered a promising differentiating characteristic, preferentially determined by non-invasive in vivo imaging, which may improve personalized theranostics of malignant melanoma.

## 1. Introduction

Cutaneous malignant melanoma, an aggressive tumor entity with steadily increasing incidence, tends to metastasize early and, once in a metastatic stage (stage IV), represents a fatal neoplasm with scarce therapeutic options. This results in a 5 year survival below 10% and accounts for up to 90% of skin cancer deaths despite its relatively low frequency that amounts to only 5% of all invasive skin cancer cases [1,2]. Malignant melanoma usually spreads first through the local lymphatics to regional lymph nodes and later on through the blood vascular system [3,4]. Therefore, both tumor angiogenesis and lymphangiogenesis are of outstanding importance for the progression of malignant melanoma [5,6,7,8,9]. In line with this, malignant melanoma is characterized by high vascularization and elevated expression of both vascular endothelial growth factors (VEGF) and VEGF receptors (VEGFR) which strongly correlates with melanoma progression and poor clinical outcome [4,10,11,12,13]. Therefore, the VEGF-VEGFR pathway is suggested to be a valid therapeutic target in malignant melanoma [14]. Drugs targeting VEGF and VEGFR comprise VEGF-neutralizing antibodies, eg bevacizumab (Avastin^®^, Roche, Basel, Switzerland), as well as the low molecular weight protein tyrosine kinase (VEGFR) inhibitors sorafenib (Nexavar^®^, Bayer, Zürich, Switzerland), sunitinib (Sutent^®^, Pfizer, Zürich, Switzerland), or pazopanib (Votrient^®^, Novartis, Rotkreuz, Switzerland) [15,16,17,18,19]. In general, tumor blood and lymph vasculature is not only abnormal in almost all aspects of their structure, but also in their function [20,21,22,23]. As a consequence, delivery of nutrients, oxygen, and drugs as well as clearance of products of metabolism is reduced in some regions of the tumor, resulting in high interstitial fluid pressure, low pH, and hypoxia [24]. Due to genetic and epigenetic mechanisms, tumor cells in such regions present resistance mechanisms and a more malignant phenotype [20,24]. Anti-angiogenic therapy aims at normalization rather than complete destruction of tumor vasculature in order to potentiate the effects of chemo- and radiation therapy by increased delivery of therapeutics and oxygen. Unfortunately, despite the robust anti-tumor activity of anti-angiogenic substances in preclinical tumor models, for cancer patients these compounds provide only limited benefit, even in adjuvant and combination therapy and requiring acceptance of adverse side effects [14,25,26,27,28,29,30]. Therefore, new therapeutic concepts, especially for malignant melanoma, are urgently needed.

Receptor tyrosine kinase EphB4 and its preferred natural ligand EphrinB2 are transmembrane proteins and binding of EphrinB2 to EphB4 initiates downstream signaling in both the receptor- and the ligand-bearing cell, designated as (EphB4) forward and (EphrinB2) reverse signaling, respectively [31]. Bidirectional EphB4 EphrinB2 signaling was shown to be a key regulator of VEGFR-2 and VEGFR-3 internalization and signaling, thereby controlling both VEGF mediated angiogenesis and lymphangiogenesis during embryonic development [32,33,34,35,36,37,38,39,40,41]. Moreover, there are a number of reports demonstrating the importance of EphB4 and EphrinB2 for postnatal angiogenesis, in particular for tumor angiogenesis and its effect on tumor growth [42,43]. Interestingly, in colorectal cancer patients receiving bevacizumab therapy, EphB4 has been identified as a predictive biomarker, with an increased EphB4 expression in non-responders [44]. This may be of particular interest, since overexpression of EphB4 has been reported for several tumor entities comprising colon, breast, bladder, endometrium, and ovarian carcinomas [45,46,47,48,49,50]. With regard to malignant melanoma, reliable preclinical and clinical data are still missing to date. However, in an A375 melanoma xenograft model EphB4 overexpression has been found to be responsible for resistance to cisplatin, a DNA-damaging compound commonly used in the treatment of malignant melanoma [51]. Using a combination of non-invasive small animal positron emission tomography (PET), magnetic resonance imaging (MRI), and optical (fluorescence) imaging in vivo, together with ex vivo imaging modalities, we systematically investigated the functional relevance of EphB4 overexpression in A375 melanoma xenografts as model. We demonstrated EphB4 to be a positive regulator of tumor growth, but a negative regulator of tumor vascularization and perfusion, resulting in increased tumor hypoxia. This characteristic is expected to be rather unfavorable for patients’ prognosis and therapy outcome, since tumor hypoxia substantially contributes to both chemo- and radioresistance of tumors [52]. Therefore, functional expression of EphB4 may represent both a promising biomarker and theranostic target in malignant melanoma.

## 2. Results

### 2.1. EphB4 Expression in Transfected A375 Melanoma Cells and Corresponding Tumor Xenografts

**Figure 1:**
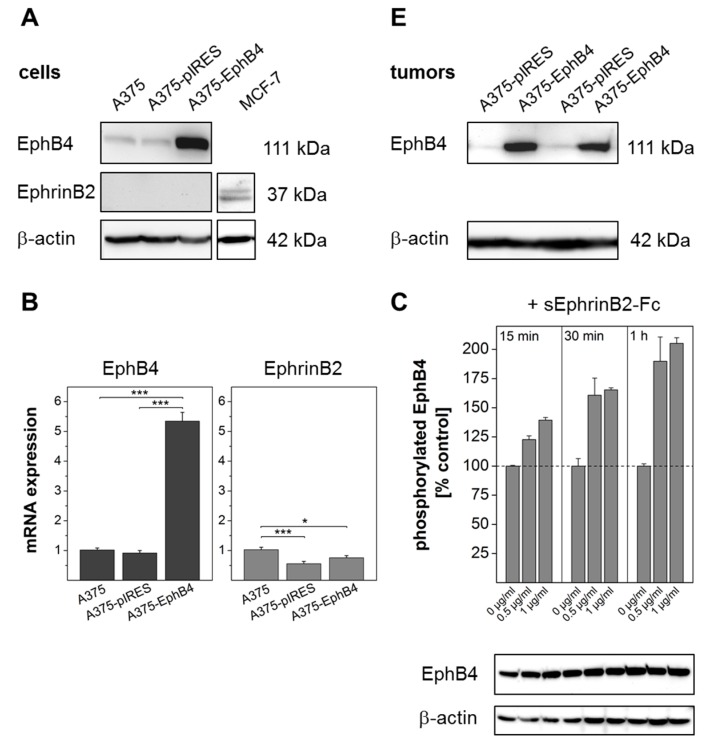

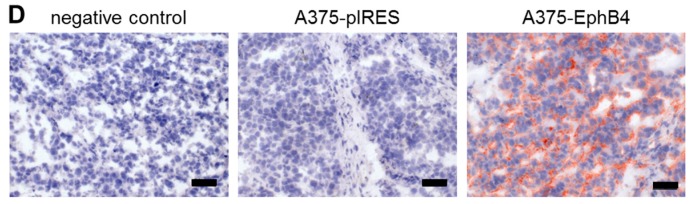
EphB4 expression in transfected A375 melanoma cells and corresponding tumor xenografts. Western blot analysis of EphB4 and its ligand EphrinB2 in A375, A375-pIRES, and A375-EphB4 whole cell lysates (**A**) and tumor lysates (**E**). Anti-β-actin served as loading control. (**B**) Relative mRNA expression of EphB4 and EphrinB2 in A375, A375-pIRES, and A375-EphB4 cells, analyzed by quantitative real-time RT-PCR was normalized to the constitutive expression level of β-actin and to expression level in wild-type A375 melanoma cells using the delta delta CT method (2^−∆∆ct^) resulting in a value of 1 for A375 cells. Values represent mean ± SEM of at least three independent experiments each performed in triplicate (* *p* < 0.05, *** *p* < 0.001). (**C**) EphB4 phosphorylation was analyzed by pEphB4-ELISA in whole cell lysates of A375 EphB4 cells incubated with different concentrations of sEphrinB2-Fc (0; 0,5; 1 µg/mL) for 15, 30, and 60 min. Values represent mean ± SD from one of at least three independent experiments, each performed in duplicate. To rule out influence of sEphrinB2 on total EphB4 protein amount, EphB4 was analyzed in the same cell lysates by western blot analysis (figure shows one representative blot out of three independent experiments performed in duplicate) ranging from 92% to 112% of control as calculated after densitometric analysis. Anti β-actin served as loading control. (**D**) Immunohistochemical detection of EphB4 in acetone-fixed cryosections of A375 pIRES and A375 EphB4 tumor xenografts using goat anti EphB4 antibody. Sections stained without the primary antibody served as negative control. Scale bar 50 µm.

Increased expression of EphB4 was confirmed by western blot analysis (Figure 1A) and quantitative real-time RT-PCR (Figure 1B) in A375 melanoma cells transfected with pIRES2-AcGFP1-EphB4 (A375-EphB4), but not in A375 cells transfected with mock plasmid (A375-pIRES) or non-transfected control (A375). To ensure that overexpressed EphB4 is functional and can be activated by its preferred ligand EphrinB2, we analyzed EphB4 tyrosine phosphorylation after incubation of A375-EphB4 cells with sEphrinB2-Fc. EphB4 tyrosine phosphorylation increased depending on EphrinB2-Fc concentration and incubation time, whereas total EphB4 protein amount remained nearly constant (Figure 1C). Endogenous expression of EphrinB2, analyzed by quantitative PCR, was slightly reduced in transgenic cell lines, but did not differ between A375-EphB4 and mock cells (Figure 1B). On protein level, EphrinB2 could not be detected in any of the investigated A375 melanoma cells, but in breast cancer cell line MCF-7 serving as positive control (Figure 1A). This is consistent with data published elsewhere showing abundant EphrinB2 mRNA expression but no detectable EphrinB2 protein in A375 wild type cells [53], which indicates an EphrinB2 protein level lower than the detection level of the available antibodies. However, at least a very low EphrinB2 protein expression by A375 melanoma cells cannot be ruled out. To examine whether EphB4 overexpression is retained during growth of tumor xenografts, EphB4 expression was analyzed in resected tumors at least 14 d post tumor cell injection. Both, immunohistochemistry and western blot analysis confirmed an increased EphB4 expression level in A375-EphB4 tumors in comparison to A375-pIRES tumors (Figure 1D,E).

### 2.2. EphB4 Promotes Growth of A375 Melanoma Cells and Xenografts

**Figure 2:**
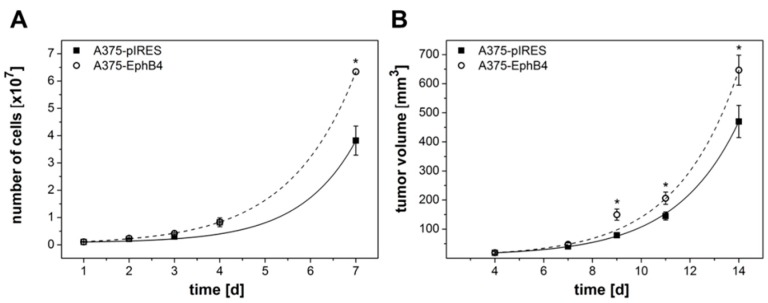

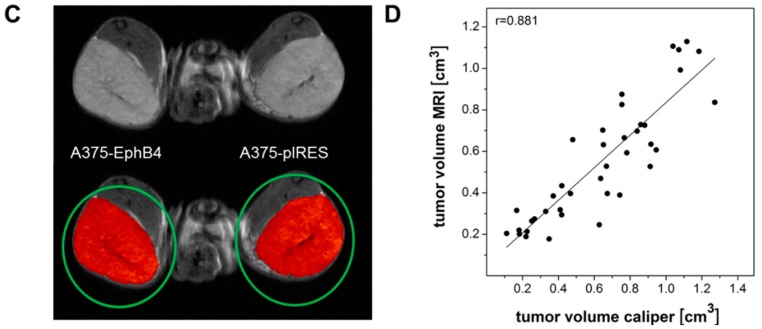
Overexpression of EphB4 in A375 melanoma cells increases cell proliferation and growth of corresponding tumor xenografts. (**A**) Cells were cultivated in 6-well plates and number of viable cells was determined after trypsinization using a CASY^®^Model TT cell counter. Values represent mean ± SEM from one of at least three independent experiments, each performed in quadruplicate (* *p* < 0.05). (**B**) Tumor size was monitored thrice a week by caliper measurement and tumor volume was calculated using the formula V = π/6 × (tumor length × tumor width^2^). Values represent mean ± SEM from three independent experiments with a total of 49 mice (* *p* < 0.05). (**C**) In addition to caliper measurement, tumor volume was determined in a subset of mice by MRI. Using the ROVER software, 3-dimensional regions of interest (ROIs, colored in red) were determined within masks (green circles) around the tumors by thresholding MRI data within these masks and manual adjustment if necessary. (**D**) Tumor volumes determined by MRI and ROVER software analysis correlate with tumor volumes measured by caliper (*r* = 0.881, *p* < 0.001).

Overexpression of EphB4 in A375 melanoma cells slightly but significantly increased both cell proliferation in vitro and tumor growth in vivo (Figure 2A,B). At day 14 post tumor cell injection A375-EphB4 tumors reached a mean volume of 647 ± 52 mm^3^ in comparison to 470 ± 55 mm^3^ for A375-pIRES tumors. Since caliper measurement sometimes may be incorrect due to tumor shapes differing from ellipsoid or due to swelling, in a subset of mice we verified tumor volume at day 14–18 post tumor cell injection using MRI and ROVER software (Figure 2C). Thereby, we observed a significant correlation between tumor volumes measured by caliper and MRI (Figure 2D, *r* = 0.881, *p* < 0.001). As a consequence, for all further experiments tumor volume was determined by caliper measurement.

### 2.3. EphB4 Decreases Perfusion and Vascularization of A375 Melanoma Xenografts

**Figure 3:**
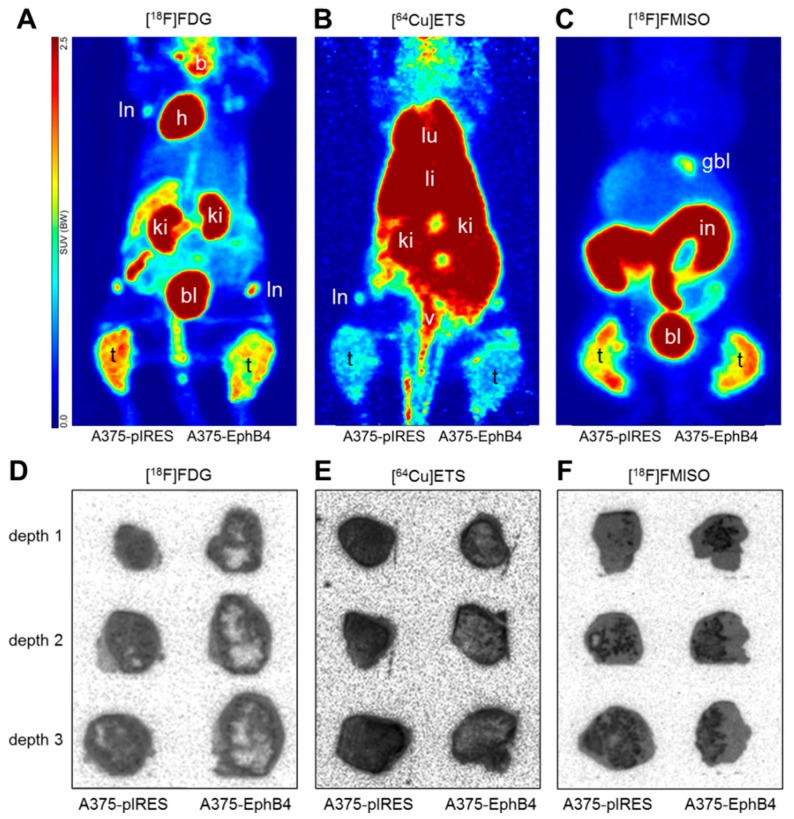

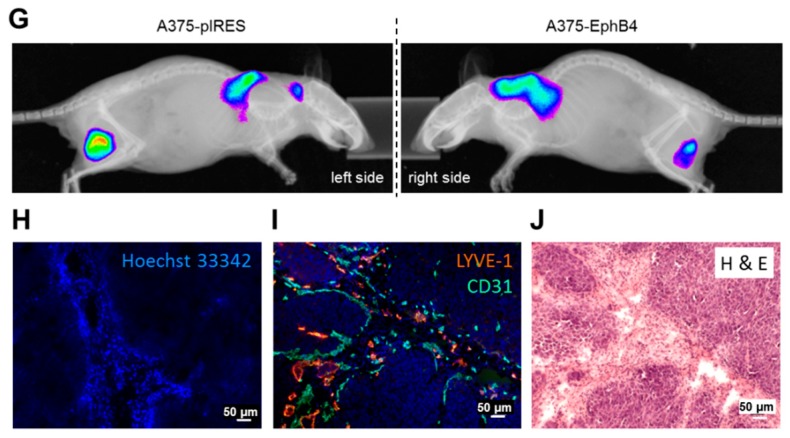
Representative images of PET, radioluminography, in vivo fluorescence imaging, and ex vivo (fluorescence) microscopy of A375-pIRES/-EphB4 tumor-bearing NMRI nu/nu mice and tumor cryosections. After intravenous injection [^18^F]FDG, [^64^Cu]Cu-ETS, and [^18^F]FMISO clearly accumulate in A375-pIRES and A375-EphB4 tumors and can be visualized by small animal PET (**A**–**C**) and subsequent radioluminography (**D**–**F**). (**A**–**C**) PET data are illustrated as maximum intensity projection (MIPs) of tracer-dependent frame times ([^18^F]FDG, 30–60 min p.i.; [^64^Cu]Cu-ETS, 0–30 min p.i.; [^18^F]FMISO, 180–240 min p.i.). (**D**–**F**) Subsequent to PET imaging, A375-pIRES/-EphB4 tumors were resected and distribution of PET tracers was analyzed by radioluminography in tumor cryosections (18 sections each in 3 tumor depth). (**G**) In vivo fluorescence imaging of A375-pIRES/-EphB4 tumor-bearing NMRI nu/nu mice 24 h post i.v. injection of AngioSense (750/790 nm). (**H**) Ex vivo fluorescence microscopy of A375-pIRES/-EphB4 tumor cryosections following i.v. injection of 30 mg/kg Hoechst 33342 exactly 1 min before sacrifice of mice. (**I**) Tumor cryosections were stained with CD31 and LYVE-1 specific primary antibodies as well as Alexa Fluor 488- and Alexa 546-conjugated secondary antibodies to determine blood and lymph vessel markers CD31 (colored in green) and LYVE-1 (colored in orange), respectively. (**J**) Subsequent to fluorescence microscopy, tumor cryosections were stained with H&E to get detailed data on tumor tissue, tumor stroma, and adjacent mouse tissue. Abbreviations: b, brain; bl, bladder; gbl, gall bladder; h, heart; in, intestine; li, liver; ln, lymph node; lu, lung; t, tumor; ki, kidney; v, vein.

**Figure 4:**
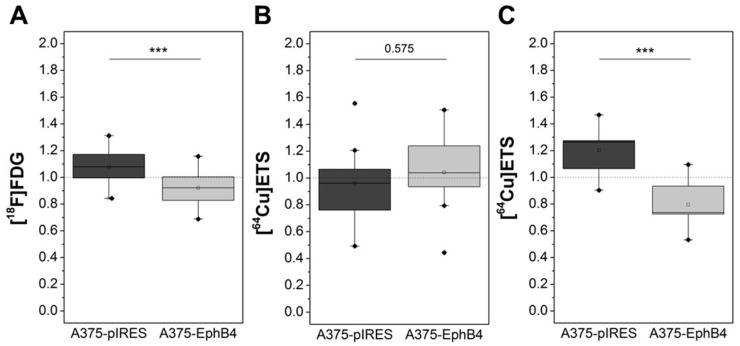
Overexpression of EphB4 in A375 melanoma cells decreases perfusion of tumor xenografts as determined by small animal PET imaging and subsequent ex vivo radioluminography after i.v. injection of [^18^F]FDG and [^64^Cu]Cu-ETS. A375-pIRES/-EphB4 tumor-bearing NMRI nu/nu mice were i.v. injected with [^18^F]FDG (**A**) or [^64^Cu]Cu-ETS (**B**) and tracer kinetics were determined by PET. Perfusion-associated rate constant K1 was analyzed by graphical evaluation of PET data according to Patlak. (**C**) Subsequent to PET imaging, A375-pIRES/-EphB4 tumors were resected and distribution of [^64^Cu]Cu-ETS was analyzed by radioluminography in tumor cryosections (18 sections each in 3 tumor depth). Mean radioluminographic intensity per mm^2^ was analyzed using ROVER software. (**A**–**C**) Results of tracer kinetic analysis and radioluminography are demonstrated as relative values (eq. 2 × K1_pIRES_/(K1_pIRES_ + K1_EphB4_; *** *p* < 0.001).

Perfusion of A375 melanoma xenografts was investigated by dynamic small animal PET using the widely available PET tracer [^18^F]FDG (Figure 3A) [54]. Since [^18^F]FDG is only a surrogate marker for perfusion, in a subset of nine mice we further used the previously little noticed experimental perfusion tracer [^64^Cu]Cu-ETS, showing a microsphere-like behavior (Figure 3B) [55]. Tracer kinetic analysis revealed a significantly decreased perfusion-associated rate constant K1 in A375-EphB4 tumors in comparison to mock tumors for [^18^F]FDG, but not for [^64^Cu]Cu-ETS (Figure 4A,B). Nevertheless, radioluminographic analysis of [^64^Cu]Cu-ETS tumor accumulation (Figure 3E) clearly confirmed a decreased perfusion of A375-EphB4 tumors in comparison to mock tumors (Figure 4C). This apparent discrepancy can be explained, on the one hand, by higher spatial resolution of radioluminography in comparison to PET imaging and, on the other hand, by inferior suitability of copper-64 in comparison to fluorine-18 as a PET nuclide due to higher positron energy (max β+ energy, 0.65  vs. 0.64 MeV), resulting in higher positron range (max linear range in H_2_O, 2.9 vs. 2.4 mm), and lower positron decay rate (18% vs. 97%) of copper-64 [56]. Representative radio-luminography of [^18^F]FDG distribution in tumor sections 1 h p.i. provided some evidence for an increased volume of tumor tissue characterized by diminished glucose metabolism within A375-EphB4 tumors (Figure 3D), most likely due to augmented necrosis.

**Figure 5:**
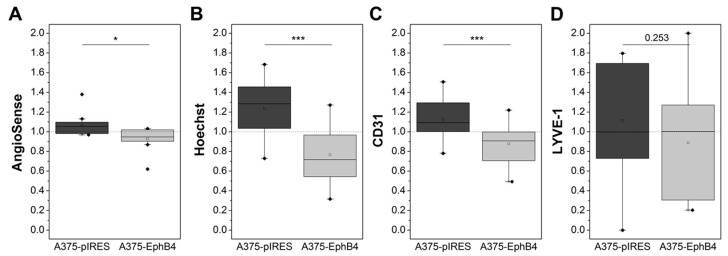
Overexpression of EphB4 in A375 melanoma cells decreases amount of functional blood vasculature in tumor xenografts as determined by in vivo fluorescence imaging, ex vivo Hoechst 33342 fluorescence microscopy, and CD31/ LYVE-1 immunohistochemistry. (**A**) In vivo fluorescence imaging of A375-pIRES/-EphB4 tumor-bearing NMRI nu/nu mice was performed 24 h post i.v. injection of AngioSense. Mean fluorescence intensity of AngioSense (750/790 nm) was divided by mean fluorescence intensity of reference channel (430/535 nm). (**B**) Mice were sacrificed exactly 1 min post i.v. injection of 30 mg/kg Hoechst 33342. Tumor cryosections were analyzed by fluorescence microscopy to determine Hoechst 33342 positive tumor fraction. (**C** and **D**) Blood vessel marker CD31 and lymph vessel marker LYVE-1 were stained in tumor cryosections by immunohistochemistry. Tumor sections were analyzed by fluorescence microscopy to determine CD31 and LYVE-1 positive tumor fraction. (**A**–**D**) Results of in vivo fluorescence imaging as well as fluorescence microscopy are demonstrated as relative values (eq. 2 × CD31_pIRES_/(CD31_pIRES_ + CD31_EphB4_); * *p* < 0.05, *** *p* < 0.001).

Intravenous injection and fluorescence imaging of the near-infrared fluorescent in vivo blood pool imaging agent AngioSense (Figure 3G, Figure 5A) gave first indications that diminished tumor vascularization may be the cause for decreased perfusion of A375-EphB4 tumors (Figure 4A), as observed by small animal PET and radioluminography. To get higher-resolution information about regional distribution and extent of functional tumor vasculature, fluorescence dye Hoechst 33342 was administered i.v. 1 min before sacrifice of mice. Quantification of Hoechst 33342-positive tumor area (Figure 3H) confirmed a reduced amount of perfused blood vessels in A375-EphB4 tumors (Figure 5B). Differentiated consideration of CD31 positive blood vessels and LYVE-1 positive lymph vessels (Figure 3I) revealed that blood vasculature is, in contrast to lymph vasculature, significantly diminished in A375-EphB4 tumors (Figure 5C,D). However, there was a broad intertumoral variability in lymph vessel density just leaving a trend towards reduced lymph vessel density in A375-EphB4 tumors.

### 2.4. EphB4 Increases Hypoxia of A375 Melanoma Xenografts

**Figure 6:**
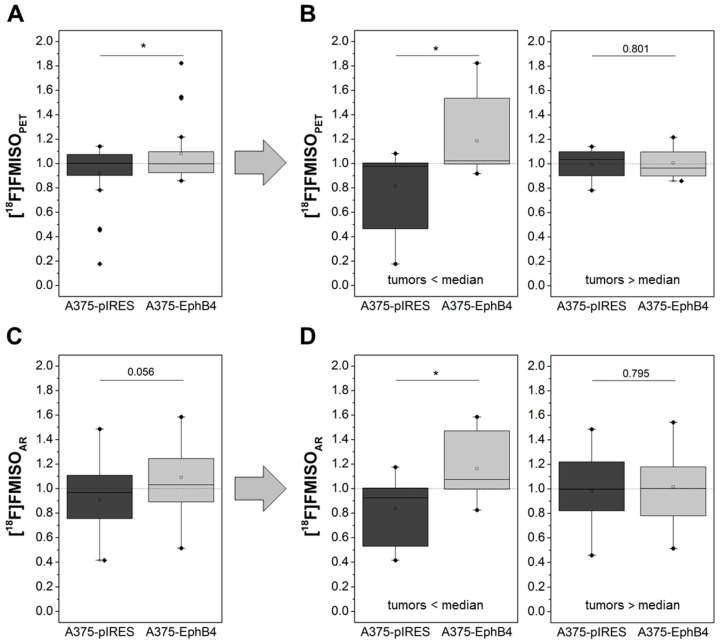
Overexpression of EphB4 in A375 melanoma cells increases hypoxia of tumor xenografts as determined by small animal PET imaging and subsequent ex vivo radioluminography after i.v. injection of [^18^F]FMISO. (**A**) A375-pIRES/-EphB4 tumor-bearing NMRI nu/nu mice were i.v. injected with [^18^F]FMISO and tracer kinetics were determined by PET. Km value was determined by graphical evaluation of PET data according to Patlak. (**C**) Subsequent to PET imaging, A375 pIRES/-EphB4 tumors were resected and distribution of [^18^F]FMISO was analyzed by radioluminography (AR) in tumor cryosections (18 sections each in three tumor depths). For each tumor, [^18^F]FMISO positive tumor fraction (hypoxic fraction) was analyzed using ROVER software. (**B**,**D**) For detailed analysis, mice were divided into two groups according to the sum of their tumor volumes and the median tumor volume sum of all mice (A375-pIRES + A375-EphB4 </> 1582.3 mm^3^) and results of tracer kinetic analysis as well as radioluminography are shown separately for these two groups of mice. All results are demonstrated as relative values (eq. 2 × K_m;pIRES_/(K_m;pIRES_ + K_m;EphB4_, * *p* < 0.05).

Hypoxia in A375 melanoma xenografts was investigated by dynamic small animal PET imaging and subsequent ex vivo radioluminography using the hypoxia tracer [^18^F]FMISO (Figure 3C,F). Graphical evaluation of PET tracer kinetics according to Patlak revealed a significantly increased Km value in A375-EphB4 tumors in comparison to mock tumors (Figure 6A). [^18^F]FMISO positive tumor fraction (hypoxic fraction), determined by radioluminography of tumor cryosections, tends to be higher in A375-EphB4 tumors than in mock tumors (Figure 6C). Since results of radioluminography narrowly missed significance, we decided to divide mice into two groups according to the sum of their tumor volumes and the median tumor volume sum of all mice (A375 pIRES + A375-EphB4 </> 1582.3 mm^3^). This differentiated consideration revealed that EphB4 significantly increases tumor hypoxia especially in smaller tumors (tumors < median), whereas in the group of larger tumors (tumors > median) EphB4 has no effect on tumor hypoxia (Figure 6D). Differentiated consideration of PET data analogous to radioluminography confirmed this result (Figure 6B).

### 2.5. EphB4 forward Signaling Is Not Responsible for Decreased Perfusion and Increased Hypoxia

**Figure 7:**
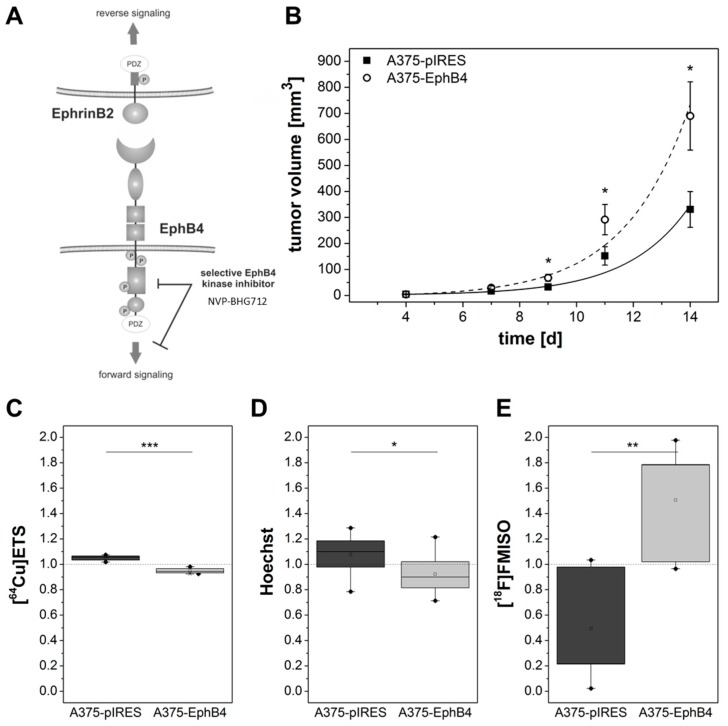
Influence of EphB4 forward signaling or EphrinB2 reverse signaling. (**A**) Interaction of EphB4 with its preferred ligand EphrinB2 can cause signaling in both EphB4 expressing cell (EphB4 forward signaling) and EphrinB2 expressing cell (EphrinB2 reverse signaling). EphB4 forward but not EphrinB2 reverse signaling can be blocked by the small molecule selective EphB4 kinase inhibitor NVP-BHG712. (**B**–**E**) From day 1 post tumor cell injection mice received weekdays 10 mg/kg NVP-BHG712. (**B**) Tumor size was monitored trice a week by caliper measurement and tumor volume was calculated using the formula V = π/6 × (tumor length × tumor width^2^). Values represent mean ± SEM from a pilot blocking experiment with a total of 14 mice (* *p* < 0.05). (**C** and **E**) Radioluminography of [^64^Cu]Cu-ETS and [^18^F]FMISO distribution in A375-pIRES/-EphB4 tumors sections was performed in 3 tumor depths with 9 sections each depth. For [^64^Cu]Cu-ETS and [^18^F]FMISO mean radioluminographic intensity per mm^2^ and [^18^F]FMISO positive tumor fraction (hypoxic fraction), respectively, were analyzed using ROVER software. (**D**) Mice were sacrificed exactly 1 min after i.v. injection of 30 mg/kg Hoechst 33342. Tumor sections were analyzed by fluorescence microscopy to determine Hoechst 33342 positive tumor fraction. (**C**–**E**) Results of radioluminography and Hoechst 33342 fluorescence microscopy are demonstrated as relative values (eq. 2 × Hoechst_pIRES_/(Hoechst_pIRES_ + Hoechst_EphB4_; * *p* < 0.05, ** *p* < 0.01, *** *p* < 0.001).

Either EphB4 forward signaling or EphrinB2 reverse signaling (Figure 7A) can initiate decreased perfusion and vascularization as well as increased hypoxia in A375-EphB4 tumors. In order to discriminate these two signaling pathways, we performed a pilot experiment with the small molecule EphB4 specific kinase inhibitor NVP-BHG712 (ED50 = 25 nM) as blocking agent [57]. Here we focused on analysis of ex vivo radioluminography after [^18^F]FMISO and [^64^Cu]Cu-ETS injection as well as on Hoechst 33342 fluorescence microscopy since these in this study appeared to be as most informative regarding tumor hypoxia, perfusion, and vascularization. Of importance, blocking of EphB4 forward signaling in A375-EphB4 tumors did not substantially influence perfusion and vascularization as well as development of hypoxia (Figure 7C–E). Moreover, blocking of EphB4 forward signaling did not alter A375-EphB4 tumor growth (Figure 7B).

## 3. Discussion

In this study, we demonstrate EphB4 to be a promotor of tumor growth and hypoxia and an inhibitor of tumor vascularization and perfusion in A375 melanoma xenografts in vivo. In the human melanoma cell model used overexpression of EphB4 resulted in increased cell proliferation in vitro and tumor growth in vivo. In a previous study that predominantly was performed for radiopharmacological characterization of a novel EphB4 targeting radioligand in 12 mice no substantial difference in tumor growth between EphB4 and mock transfected A375 tumors was observed [58]. In our present study comprising 49 mice we observed an increased tumor growth of EphB4 overexpressing melanoma xenografts, which is in line with the increased proliferation of A375 EphB4 cells in vitro.

The influence of EphB4 on tumor growth in experimental studies is contradictory and seems to be highly dependent on the cellular context, especially on co-expression of its preferred ligand EphrinB2. In this regard, EphB4 was shown to promote tumor growth only when melanoma cells co-express EphrinB2 [59,60,61,62,63]. Therefore, we analyzed EphrinB2 expression in our wild type and transgenic A375 melanoma cells. Both in the present and a previous study, we were able to detect EphrinB2 on mRNA level but not on a protein level [53]. This is consistent with observations made by Martiny-Baron et al. and Heroult et al. [64,65]. Assuming a very low or absent EphrinB2 protein expression in A375 cells, we would have expected tumor growth suppression as a consequence of EphB4 overexpression, comparable to the effect observed for overexpression of sEphB4, a soluble form of the EphB4 receptor only sufficient to activate EphrinB2 reverse signaling [64]. Instead we observed an increased tumor growth in A375-EphB4 tumor xenografts. This might be explained by the fact that even very low EphrinB2 protein levels in A375 cells could be sufficient enough for tumor promoting reverse signaling. Moreover, activation of EphrinB2 reverse signaling on adjacent cells, eg endothelial cells, is also important for tumor suppressive effects of EphB4 [64]. Therefore, a detailed differentiation of EphrinB2 expression in tumor and tumor stroma of A375 xenografts should be addressed in further investigations. However, since the used EphrinB2 antibody reacts with EphrinB2 of both human and mouse origin the present results suggest overall very low EphrinB2 protein levels in tumor lysates, comprising both human tumor cells and murine stroma cells. On the other hand, clinical and experimental data support that increased EphrinB2 expression in human melanoma is associated with tumor progression and metastasis [66].

In the above-mentioned studies, EphB4 induced modulation of tumor growth was accompanied by changes in tumor vasculature, but analysis of tumor vasculature therein was limited to morphological parameters like vessel density or vessel diameter [60,61,64]. Unfortunately, tumor vessel quantification does not always correlate with the functional status of tumor vasculature and functionality of vessels rather than morphological parameters may determine disease prognosis in melanoma and other cancers [14,67]. PET, by contrast, allows for non-invasive characterization of parameters like tumor perfusion and hypoxia, which may better reflect the true functional status of a tumor vasculature. Therefore, we investigated perfusion of A375 melanoma xenografts by dynamic small animal PET imaging using [^18^F]FDG and [^64^Cu]Cu-ETS, representing a surrogate marker for perfusion and a previously little noticed experimental perfusion tracer, respectively [54,55,68,69].

Tracer kinetic analysis revealed a significantly decreased perfusion-associated rate constant K1 in A375-EphB4 tumors in comparison to mock tumors for [^18^F]FDG, but not for [^64^Cu]Cu-ETS. Nevertheless, ex vivo radioluminography of [^64^Cu]Cu-ETS tumor accumulation clearly confirmed a decreased perfusion of A375-EphB4 tumors in comparison to mock tumors. To sum up, combination of in vivo PET imaging with ex vivo radioluminography enabled us to demonstrate a negative effect of EphB4 overexpression on perfusion of A375 melanoma xenografts. Subsequent fluorescence imaging using the near-infrared in vivo blood pool-imaging agent AngioSense [70,71] gave first indications that decreased perfusion of A375-EphB4 tumors may be caused by a diminished tumor vascularization. In order to get information at higher resolution about regional distribution and the extent of functional tumor vasculature, fluorescence dye Hoechst 33342 was administered intravenously 1 min before sacrifice of mice. Due to its fast distribution with blood stream, its limited diffusion across cell layers, and its quick DNA binding potential, Hoechst 33342 enables staining of nuclei in cells lining a perfused blood vessel, and thereby is an indicator for functional (perfused) blood vessels [72]. Quantification of Hoechst 33342- and CD31-positive tumor area confirmed a reduced amount of (functional) blood vessels in A375 EphB4 tumors in comparison to mock tumors. Incidentally, the decreased perfusion and vascularization of A375-EphB4 melanoma xenografts may be an explanation for failure of EphB4 targeted imaging approaches by our group [58]. As a consequence of reduced tumor perfusion, a novel and maybe appropriate radiotracer might be under-valuated due to inaccessibility of the target EphB4 for the PET tracer.

Influence of EphB4 on tumor angiogenesis is not surprising since EphB4 receptor and its preferred ligand EphrinB2 are essential regulators of embryonic blood and lymph vascular morphogenesis [31,32,33,34,35,36,37,73]. Nevertheless, our results are in clear contrast to recently published results using carcinoma models like breast and colorectal cancer, where overexpression of EphB4 promotes tumor growth by stimulating tumor angiogenesis through increased EphrinB2 reverse signaling [42,43]. In this situation, disruption of EphB4-EphrinB2 interaction may represent a potential opportunity for anti-angiogenic cancer therapy, and on the other hand, a more efficient vasculature due to increased EphrinB2 signaling may at least facilitate delivery of anticancer drugs to tumor tissue [42,43]. The situation observed in the A375-EphB4 melanoma model, by contrast, would have far-reaching consequences for tumor therapy, since decreased tumor perfusion hampers accessibility of a tumor for therapeutics like anticancer drugs. This may, in part, be an explanation for the EphB4-mediated cisplatin resistance of A375 melanoma xenografts [51].

Moreover, using [^18^F]FMISO we demonstrated increased hypoxia in A375-EphB4 tumor xenografts in comparison to mock tumors. [^18^F]FMISO is taken up by cells and its cellular trapping depends on the intracellular oxygen concentration [74,75]. In particular, by in vivo PET imaging it became evident that EphB4 overexpression increased hypoxia especially in ‘smaller’ tumors (sum of A375-pIRES and A375-EphB4 tumor volumes < 1582.3 mm^3^) which were normally not yet hypoxic. The missing effect in ‘larger’ tumors (sum of A375-pIRES and A375-EphB4 tumor volumes > 1582.3 mm^3^) may be explained by diminished [^18^F]FMISO trapping in necrotic regions, when hypoxia is omitted on behalf of necrosis in fast growing tumors. Hypoxia further hampers tumor therapy, because it increases therapy resistance of tumor cells to both radiation and chemotherapeutic agents [52,75,76,77,78]. Moreover, hypoxia has been shown to increase aggressiveness as well as metastasis of cancer cells [52].

Since lymph vasculature is an important route for tumor cell metastasis [8,9] and lymphangiogenesis was the most sensitive prognostic indicator for lymph node metastasis of cutaneous melanoma [6,7], we investigated if EphB4 also influences the amount of lymph vessels in the A375 melanoma xenograft model. For this purpose, we quantified vessels positive for lymphatic vessel endothelial hyaluronan receptor-1 (LYVE-1), the earliest as well as most widely used marker for lymph endothelial cells [79,80]. By contrast to CD31 positive blood vasculature, amount of LYVE-1 positive lymph vasculature was not significantly influenced by EphB4 overexpression in the A375 melanoma model, perhaps because of the broad intertumoral variability in lymphatic vessel density.

Bidirectional EphB4-EphrinB2 signaling, and in particular EphrinB2 reverse signaling through its PDZ domain, has been shown to be essential for appropriate VEGF induced VEGFR-2 and VEGFR-3 internalization and signaling, and thereby to be a key regulator of (lymph)angiogenesis [40,41]. Nevertheless, up to now it is unclear if EphB4 is only the trigger for EphrinB2 reverse signaling or if there is a comparable level of importance for EphB4 forward signaling [36,39,40,41,81]. Interestingly, EphB4 but not EphrinB2 has been identified as a predictive biomarker for therapy response in colorectal cancer patients receiving bevacizumab, with an increased EphB4 expression in non-responders [44]. Moreover, combination of bevacizumab with inhibitory EphB4 specific monoclonal antibodies targeting the extracellular fibronectin-like domains significantly inhibited growth of HT-29 colon carcinoma xenografts more than monotherapy [82,83]. Decreased perfusion and vascularization as well as increased hypoxia in A375-EphB4 tumors can be initiated by either EphB4 forward signaling or EphrinB2 reverse signaling. In order to discriminate these two signaling pathways, we performed a pilot experiment with the small molecule EphB4 specific kinase inhibitor NVP-BHG712 (ED_50_ = 25 nM) as blocking agent [57]. NVP-BHG712 has been shown to be selective for EphB4 with some lower inhibitory potential to off-targets like EphB2, EphA2, EphB3, EphA3, c-raf, c-src, and c-Abl [57]. However, since A375-pIRES and A375-EphB4 originate from the same A375 wild-type cells they should primary differ in their EphB4 expression level. In this pilot experiment we focused on analysis of ex vivo radioluminography after [^18^F]FMISO and [^64^Cu]Cu-ETS injection as well as on Hoechst 33342 fluorescence microscopy since these in this study appeared to be as most informative regarding tumor hypoxia, perfusion, and vascularization. Of importance, blocking of EphB4 forward signaling in A375-EphB4 tumors did not substantially influence perfusion and vascularization as well as development of hypoxia. Moreover, blocking of EphB4 forward signaling did not alter A375-EphB4 tumor growth. Taken together, results of this pilot blocking experiment did not support major or exclusive involvement of kinase domain mediated EphB4 forward signaling but support possible involvement of EphrinB2 reverse signaling despite of the obviously very low EphrinB2 protein level in this tumor xenograft model.

## 4. Materials and Methods

### 4.1. Generation of EphB4 Overexpressing A375 Melanoma Cells

Generation of expression constructs as well as stable transfection of human A375 melanoma cells was performed as described elsewhere [58]. In brief, A375 melanoma cells were transfected via nucleofection using Amaxa^®^ Nucleofector technology (Lonza, Cologne, Germany) and the expression constructs pIRES2 AcGFP1-EphB4 (full-length EphB4) or pIRES2-AcGFP1 (mock plasmid). Cells are designated as A375 (non-transfected control), A375-pIRES (mock-transfected), and A375-EphB4 (EphB4-overexpressing). Stable expression was verified by flow cytometry (GFP expression) as well as quantitative RT-PCR and western blot analysis (EphB4 expression).

### 4.2. Flow Cytometry

For flow cytometric analysis, cells were trypsinized, washed, and immediately analyzed for their GFP expression using a FACSCalibur instrument (BD, Franklin Lakes, NJ, USA) as published elsewhere [58].

### 4.3. RNA Extraction and Quantitative Real-Time Reverse Transcription-PCR

Total RNA extraction as well as quantitative real-time reverse transcription-PCR (RT-PCR) starting with 100 ng RNA was performed as described elsewhere [53,84]. Relative mRNA expression of EphB4 and EphrinB2 was normalized to the constitutive expression level of β-actin and to expression level in wild-type A375 melanoma cells using the delta delta CT method (2^−∆∆ct^ ) resulting in a value of 1 for A375 cells.

### 4.4. Immunoblotting

Preparation of protein extracts from subconfluent cell cultures or resected tumors using RIPA buffer as well as western blot analysis was performed as described elsewhere [58]. In brief, 80 µg of proteins were separated in a 8.5% *v*/*v* sodium dodecyl sulfate polyacrylamide gel electrophoresis (SDS-PAGE) and transferred to a PVDF membrane (Fisher Scientific, Schwerte, Germany) using a semi-dry transfer system (Bio-Rad, Munich, Germany). After blocking with 5% *w*/*v* skimmed milk for at least 1 h, PVDF membrane was incubated with goat anti-human EphB4 IgG (R&D, AF3038; 0.2 µg/mL) or rabbit anti EphrinB2 IgG (Novus Biologicals, Littleton, CO, USA, NBP1-48551; 3.1 µg/mL) for 2 h at room temperature and afterwards overnight at 4 °C. Membranes were washed three times and incubated with the appropriate horseradish peroxidase coupled secondary antibodies (rabbit anti-goat IgG, Sigma Aldrich, Taufkirchen, Germany; A5420; goat anti rabbit IgG, Sigma Aldrich, A0545) for 1 h at room temperature. Protein bands were detected with Super Signal West Pico, Dura, or Femto Chemiluminescent Substrate (Fisher Scientific) and the MF-ChemiBis 3.2 imaging system (Biostep, Burkhardtsdorf, Germany). To verify equal protein loading, membranes were stripped and reprobed with mouse anti β-actin IgG (Sigma Aldrich, A5316) and horseradish peroxidase coupled rabbit anti-mouse IgG (Sigma Aldrich, A9044).

### 4.5. Phospho-EphB4 ELISA

EphB4 receptor phosphorylation was determined using the human phospho-EphB4 (pEphB4) DuoSet IC (R&D, Wiesbaden, Germany) as described elsewere in detail [58]. In brief, A375 EphB4 cells were cultured in 6 cm petri dishes for 24 h. Afterwards, cells were incubated with recombinant mouse Ephrin-B2 (Arg27 Ala227) Fc chimera protein (designated as sEphrinB2-Fc; R&D, 496-EB-200; 0, 0.5 or 1 µg/mL) for 15 min, 30 min, or 1 h. Cellular protein extracts were obtained and used for pEphB4 ELISA (300 µg/ sample) according to manufacturer’s instructions for pEphB4 DuoSet IC (R&D) and described in detail elsewhere [58]. In parallel, total EphB4 protein amount was analyzed by western blot analysis. Densitometric analysis of western blots was performed via the 1D gel analysis software Total Lab (Total Lab Limited, Newcastle upon Tyne, UK) as described elsewhere [58].

### 4.6. Cell Culture and Proliferation Assay

Human metastatic melanoma cell line A375 (LGC Standards, ATCC^®^ CRL-1619™, Wesel, Germany), transgenic A375 cells, and human breast adenocarcinoma cell line MCF-7 (LGC Standards, ATCC^®^ HTB 22™) were cultured in Dulbecco´s Modified Eagle Medium (DMEM) supplemented with 10% *v*/*v* fetal bovine serum (FBS) and 1% *v*/*v* penicillin/streptomycin (PenStrep) (all reagents from Biochrom, Berlin, Germany) at 37 °C in a humidified atmosphere with 5% CO_2_. Transgenic cells were further cultured with 1.2 mg/mL G418 sulfate (Biochrom). All cells were routinely tested for mycoplasma contamination using Venor^®^GeM Mycoplasma Detection Kit (Minerva Biolabs, Berlin, Germany). For proliferation assay, 5 × 10^4^ A375-pIRES and A375-EphB4 cells were seeded into 6 well plates. After 1, 2, 3, 4, and 7 days cells were trypsinized and the number of viable cells was determined using a CASY^®^Model TT.

### 4.7. Animals and Generation of A375 Melanoma Xenografts

All animal experiments were carried out according to the guidelines of the German Regulations for Animal Welfare. The protocols were approved by the local Ethical Committee for Animal Experiments (AZ 24D-9168.11-4/2007-2, AZ 24-9168.11-4/2012-1 and AZ 24-9168.21-4/2004-1). Generation of tumor xenografts was performed as described elsewhere [58]. In brief, NMRI nu/nu mice were subcutaneously injected with 5 × 10^6^ A375-pIRES and A375-EphB4 cells, each in 100 μL 0.9% *v*/*v* NaCl, into the left and right hind leg, respectively. Tumor size was monitored three times a week by caliper measurements and tumor volume was calculated using the formula V = π/6 × (tumor length × tumor width^2^). Tumor-bearing mice were included into the experiments about 14–18 days post tumor cell injection, when tumors reached a volume of at least 400 to 700 mm^3^.

### 4.8. Blocking Experiments with EphB4 Kinase Inhibitor NVP-BHG712

The small molecule specific EphB4 kinase inhibitor NVP-BHG712 [57] (Sigma-Aldrich) was dissolved in 1-methyl-2-pyrrolidone (NMP) with a concentration of 15 mg/mL. For oral administration, NMP-NVP-BHG712 solution was diluted with PEG300 to a final concentration of 1.5 mg NVP-BHG712 /mL (10% *v*/*v* NMP-NVP-BHG712 and 90% *v*/*v* PEG300). Using administration by gavage of 6.7 mL/kg body weight, corresponding to 10 mg NVP-BHG712 /kg body weight, once a day (weekdays only) mice received NVP-BHG712 from day 1 post tumor cell injection until end of the experiment.

### 4.9. Magnet Resonance Imaging

In addition to caliper measurement, in a subset of mice tumor volume was determined by magnetic resonance imaging (MRI) using a 7 Tesla small animal scanner (BioSpec^®^ 70/30, Bruker, Ettlingen, Germany) with a T2-weighted Turbo Rapid Acquisition with Relaxation Enhancement (TRARE) measuring sequence [85,86]. In the present study echo and repetition time were 12 and 7080 ms, respectively. Resolution was 156 µm in x-y-direction and, as determined by slice thickness, 500 µm in z direction. Quantification of tumor volume was performed by the software ROVER version (ABX GmbH, Radeberg, Germany) using a fixed threshold for signal intensity with manual adjustment.

### 4.10. Positron Emission Tomography

Dynamic positron emission tomography (PET) was performed with the three tracers 2-deoxy-2-[^18^F]fluoroglucose ([^18^F]FDG), 1-(2-nitro-imidazolyl)-3-[^18^F]fluoro-2-propanol ([^18^F]FMISO), and ethylglyoxal-bis(thiosemicarbazonato)[^64^Cu]copper(II) ([^64^Cu]Cu-ETS) using microPET^®^ P4 (Siemens Preclinical Solutions, Knoxville, TN, USA) and a NanoScan PET/CT scanner (Mediso, Budapest, Hungary) as described elsewhere [86,87]. In brief, anesthetized mice were positioned and immobilized prone with their medial axis parallel to axis of the scanner. PET acquisition was started 20 s before intravenous (i.v.) infusion of the radiotracer through a needle catheter into a lateral tail vein and emission data were acquired continuously for a tracer-dependent duration ([^18^F]FDG and [^18^F]FMISO, 0-60 min p.i.; [^64^Cu]Cu-ETS, 0–30 min p.i.). Acquired data were sorted into 28–32 time frames and reconstructed as described elsewhere [86,87]. Each mouse underwent two consecutive PET scans performed at two different days (first day: 10 MBq [^18^F]FDG; second day: 30 MBq [^18^F]FMISO or 10 MBq [^64^Cu]Cu-ETS) to minimize intraindividual variability and to enable maximum possible comparability of the different PET tracers. Altogether, 24 mice were investigated with [^18^F]FDG, 22 mice with [^18^F]FMISO, and 9 with [^64^Cu]Cu-ETS. In the PET images all tumors were delineated in the late time frames (highest accumulation of the tracer) by adjusting a threshold followed by visual inspection and manual correction if necessary. Resulting regions of interest (ROIs) were transferred to all frames and time activity curves (TACs) were generated. The arterial input function (AIF) was estimated inside the vena cava. The perfusion related parameter K1 was computed by fitting a reversible and an irreversible one compartment model to the [^18^F]FDG data of the first 5 min and to the [^64^Cu]Cu-ETS data, respectively [54,55,88]. For characterization of hypoxia the metabolic uptake rate Km was determined via Patlak analysis of the [^18^F]FMISO data [89,90,91]. ROI definition was performed using the ROVER software, version 3.0.29. Tracer kinetic analysis was performed with the R language and environment for statistical computing version 3.1.2 [92]. Results of PET tracer kinetic analysis were quantified as ratio between results for A375-pIRES or A375-EphB4 tumors and the mean of both in the same mouse, eg ratio of K1 for A375-pIRES = 2 × K1_pIRES_/(K1_pIRES_ + K1_EphB4_).

### 4.11. In Vivo AngioSense and Ex Vivo Hoechst 33342 Fluorescence Imaging

In vivo fluorescence imaging of tumor angiogenesis was performed using the fluorescent in vivo blood pool imaging agent AngioSense^®^ 750 EX (PerkinElmer, designated as AngioSense, Waltham, MA, USA) and the small animal optical imaging device In Vivo Xtreme (Bruker) as described elsewhere [86]. In brief, AngioSense was dissolved in PBS to a concentration of 20 nM according to manufacturer’s instructions and mice were intraperitoneally (i.p.) injected with 4 mL/kg body weight. At 24 h p.i. fluorescence emission was measured in near-infrared (AngioSense, 750/790 nm) and reference channel (430/535 nm) with an exposure time of 4 s. For the acquisition and quantification of images Bruker Molecular Imaging software was used. To minimize quantification of unspecific auto fluorescence, fluorescence images of AngioSense channel were first divided by reference channel. Subsequently, mean fluorescence intensity within the tumors was determined. For ex vivo fluorescence imaging of tumor vascularization, mice were i.v. injected with 30 mg/kg body weight bisbenzimide H 33342 trihydrochloride (Sigma-Aldrich, designated as Hoechst 33342) exactly 1 min before sacrifice. Immediately after sacrifice of mice, tumors were resected and freezed with 20 °C cold 2-Methylbutane (Sigma Aldrich). Tumor cryosections (10 µm) were prepared using the cryomicrotom CM1850 (Leica, Wetzlar, Germany), mounted onto SuperFrostPlus object slides, and dried. Afterwards, mounted tumor cryosections were used immediately for radioluminography and later on for Hoechst 33342 fluorescence microscopy, immunohistochemistry or hematoxylin and eosin (H&E) staining.

### 4.12. Tumor Radioluminography

For tumor radioluminography ex vivo, cryosections were exposed to a radioluminographic plate for about 30 min and scanned in a BioImaging Analyzer BAS-5000 (Fuji Photo Film, Düsseldorf, Germany).

### 4.13. Immunohistochemistry and Fluorescence Microscopy

Prior to detection of EphB4, CD31, or LYVE-1 by immunohistochemistry, tumor cryosections were fixed with acetone for 10 min at −20°C. After blocking with 3% *v*/*v* H_2_O_2_ for 10 min (only for EphB4) and with 2% *v*/*v* bovine serum albumin and 0.3% *w*/*v* skimmed milk for at least 1 h, tumor sections were incubated with primary antibodies (goat anti-human EphB4 IgG, R&D, AF3038, 0.2 µg/mL; rat anti-mouse CD31 IgG, BD, 550274, 0.08 µg/mL; rabbit anti mouse LYVE 1 IgG, ReliaTech Hamburg, Germany, 705 065 003; 1:200, 1 h, room temperature), ExtrAvidin^®^-Peroxidase (Sigma Aldrich; 1:50, 30 min, room temperature), and AEC Substrate Kit (BD). Primary antibodies for CD31 and LYVE 1 were visualized by Alexa Fluor 488-conjugated goat anti-rat IgG (Thermo Fisher Scientific, Langenselbold, Germany, A11006; 10 µg/mL) and Alexa 546-conjugated donkey anti rabbit IgG (Thermo Fisher Scientific, A10040, 10 µg/mL, LYVE-1), respectively, for 1 h at room temperature and subsequent fluorescence microscopy using the microscope AxioImager.A1 (Zeiss, Jena, Germany) and software AxioVision (Zeiss). Distribution of fluorescence dye Hoechst 33342 was analyzed without further processing of tumor cryosections by fluorescence microscopy. Subsequent to fluorescence microscopy, tumor cryosections were stained with Hematoxylin and Eosin (H&E).

### 4.14. Quantitative Analysis of Radioluminography and Fluorescence Microscopy

Radioluminography and fluorescence microscopy images were analyzed using ROVER and Fiji software [93], respectively. For all markers with exception of [^64^Cu]Cu-ETS radioluminography, the percentage of positive stained area within the tumor area was quantified with exclusion of image artefacts and adjacent mouse tissue indicated by H&E staining. In case of [^64^Cu]Cu-ETS radioluminography, mean intensity within the tumor sections was quantified in order to fulfill perfusion intensity rather than perfused tumor fraction. Results are illustrated as ratio between results for A375-pIRES or A375-EphB4 tumors and the mean of both in the same mouse, eg ratio of hypoxic fraction for A375-pIRES = 2 × HF_pIRES_/(HF_pIRES_ + HF_EphB4_).

### 4.15. Statistical Analysis

All data are presented as mean ± standard error of mean (SEM) or mean ± standard deviation (SD). Results were tested for their statistically significance using ANOVA followed by Bonferroni post hoc test with significance levels set at *p*-value< 0.05 (* *p* < 0.05, ** *p* < 0.01, *** *p* < 0.001) using software OriginPro (OriginLab, Friedrichsdorf, Germany). Correlations between data are described by Spearman’s rank correlation coefficient and two-tailed significance value (* *p* < 0.05, ** *p* < 0.01, *** *p* < 0.001).

## 5. Conclusions

We have demonstrated the receptor tyrosine kinase EphB4 to be a positive regulator of tumor growth, but a negative regulator of tumor vascularization in A375 melanoma xenografts in vivo. Due to its potential influence on, particularly, tumor vascularization and hypoxia, functional expression of EphB4 should be considered as potential differentiating characteristics or biomarker of malignant melanoma, which preferentially should be determined by non-invasive imaging. The latter would gain impetus from development of specific and selective radioligands for quantitation of EphB4’s functional expression by means of PET [58,94,95]. Considering the impact of tumor vascularization and hypoxia, eg, on chemo- and radioresistance of tumors, targeted theranostics of functional EphB4 is suspected to contribute to early detection of high risk groups and to personally improve patients’ prognosis and therapy outcome, which are the major principles of melanoma control [96].
